# Patent Foramen Ovale: The Unresolved Questions

**DOI:** 10.62684/DMFZ6956

**Published:** 2024-03-05

**Authors:** Imma Forzano, Gaetano Santulli

**Affiliations:** (a) Department of Advanced Biomedical Sciences, Division of Cardiology, “*Federico II*” University, International Translational Research and Medical Education (*ITME*) Consortium, Academic Research Unit, 80131, Naples, Italy;; (b) Department of Medicine (Division of Cardiology), Wilf Family Cardiovascular Research Institute, Albert Einstein College of Medicine, New York City, 10461, NY.

**Keywords:** Cardiology, Migraine, Patent Foramen Ovale (PFO), Platypnea-orthodeoxia syndrome, Stroke, TIA

## Abstract

Patent Foramen Ovale (PFO) is a remnant of fetal circulation that could be observed in the 25% of the population worldwide. PFO is associated to numerous clinical conditions as migraines, coronary embolization, transient ischemic attacks, and stroke. The main PFO concerns are related to its correlation to stroke, in particular in young adults. Despite the impact on morbidity that PFO could have, to date there is not clear evidence about its management and treatment. In this narrative review our aim is to summarize the more recent evidence in the literature dealing with PFO, in order to provide an updated overview on this topic.

## 1. Introduction

Patent foramen ovale (PFO) is an interatrial communication essential during fetal life allowing oxygenated blood deriving from inferior vena cava to pass from right atrium to left atrium [1]. Foramen ovale usually closes spontaneously after birth with the fusion of septum primum and septum secundum but in a variable percentage of individuals there is a failure of the closure of antenatal interatrial communication resulting in a simple overlap of septum primum and septum secundum and the principle cause of right-to-left shunt (RLS) in adults [2]. Nonetheless, functional closure is usually guaranteed by the elevated pressure in left atrium in comparison to the right atrium.

PFO prevalence is of 26%−35% in adult individual in autopsies series range with a median of 26% [3]. Thus, it is estimated that 2 billion people live with persistent RLS worldwide [4]. PFO is associated to numerous clinical conditions as migraines, coronary embolization, transient ischemic attack (TIA) and stroke [5, 6]. PFO concerns are about its correlation to stroke. Inter alia, 10% of strokes occurs in people aged between 18 and 60 years. From 1990 prevalence of stroke in young is increased of >20% and the rate incidence of >15% [2]. It is estimated that approximately 25% of strokes are cryptogenic [7] while 16% are identified as stroke of undetermined cause [8]. In particular, 25% of patients with stroke of undetermined cause have PFO. PFO is implicated in 4% of ischemic strokes [9]. In fact, PFO and carotid dissection are the two most frequent cause of stroke in young adults [10]. Thus, in case of patients with stroke and diagnosis of PFO it is more appropriate to define it as PFO-associated stroke. This new classification has been proposed by Elgendy and colleagues and refers to ischemic stroke with evidence of superficial, large deep, or retinal infarcts in the presence of a medium-risk to high-risk PFO absence of other plausible stroke causes [11].

## 2. Diagnosis

PFO diagnosis is based on the direct visualization of the interatrial defect through imaging techniques: transesophageal echo (TEE), transthoracic echo (TTE), intracardiac echo (ICE) [12]. As an alternative, diagnosis could be made by indirect visualization of the defect by individuation of RLS through transcranial Doppler (TCD). The ideal method to diagnose PFO is the catheterization: visualize the guidewire passing through the interatrial defect is the best prove of PFO presence. Nevertheless, due to the invasivity of that procedure, TEE is considered the gold standard [13].

### 2.1 TEE

TEE has showed to provide more information about the characteristic of several cardiac structures and abnormalities, included PFO[14]. TEE allows the identification of PFO and the evaluation of its sizing, tunnel length and characteristic. Moreover, it is possible to differentiate accurately between intracardiac and intrapulmonary shunt [15]. Saline solution with microbubbles is used and injected through an antecubital vein. The documented passage of microbubbles through the PFO is sufficient to make diagnosis. TEE has a sensitivity of 89%. This is probably due to the patient difficulty to collaborate to increase right atrial pressure with a Valsalva maneuver to facilitate RLS increasing right atrial pressure during TEE [2].

### 2.2 TTE

To diagnose PFO with TTE is used a four chamber or subcostal window and the saline solution with microbubbles. The ideal is to perform five injections at rest and after early and late Valsalva maneuver and coughing. The patient is invited to collaborate. The visualization of microbubbles passage from right atrium to left atrium define PFO. By convention, if the passage is visualized into 3 cardiac cycles is considered to be due to an intracardiac shunt and after 3 cardiac cycles is considered intrapulmonary shunt. To grade the PFO is necessary the number of bubbles. It is considered: small, if a passage of 10 or less bubbles is observed; moderate, if a passage from 10 to 20 is observed; large, if more than 20 bubbles or an intense opacification are observed [16]. Currently, TTE sensitivity is above 80% [17].

### 2.3 TCD

TCD is a valid method to diagnose PFO. TCD has demonstrated to detect RLS with a sensitivity of 97% and a specificity of 93% when compared with TEE [13]. Nonetheless, it makes impossible to distinguish among an intracardiac shunt and a pulmonary shunt [6]. TCD consents to grade the RLS basing on microembolic signal. In particular we can distinguish: no shunt, when no microbubbles are detected; low-grade shunt, when 1 to 10 bubbles are detected; moderate-grade shunt, when 11 to 25 bubbles are detected; high-grade shunt, when more than 25 bubbles are detected. When numerous bubbles are detected and it is not possible to count them, it is defined as “curtain effect” [18]. The curtain effect is related to higher risk of cerebrovascular event [19].

### 2.4 ICE

ICE is an invasive technique that provide detailed visualization of intracardiac structures. ICE has a resolution similar to TEE. The injection of saline solution with microbubbles is made through a venous femoral access. High quality images are obtained [12]. It has been discussed that the inferior cava flow is the principal responsible of the transport of embolic material that could lead to clinical conditions related to PFO, such as stroke. Arm injection has been reported to underestimate (up to 46%) the PFO shunt, leading to a misdiagnosed large RLS [12]; nonetheless, is considered a valid technique during intraprocedural PFO closure.

## 3. Associated clinical conditions

PFO it is not a pathological condition itself [20]. Its morbidity is related to clinical manifestation that could be associated. For example, risk factors or clinical conditions predisposing to clot formation could use interatrial communication as a selective pathway for the transit of microemboli (paradoxical embolism) [21]. Such microembolic phenomena could lead to serious adverse events. Indeed, PFO is associated with several clinical syndromes.

### 3.1 Stroke

The association with embolic stroke/TIA in young adults with undetermined cause [22] is undoubtedly the main reason why concern has risen on PFO. It is estimated that approximately 25% of strokes are cryptogenic while 16% are identified as stroke of undetermined cause. 25% of patients with stroke of undetermined cause have PFO. Therefore, PFO is implicated in 4% of ischemic stroke [9]. Nonetheless several PFO characteristics have been considered as high-risk features for the onset of stroke [RLS at rest [23], RLS grade, Atrial Septum Aneurism (ASA) [24]], the specific mechanism that lead to stroke remain uncertain [25].

Currently, paradoxical embolism is the most plausible hypothesis. It consists in the presence of a venous thrombus that through circulation arrives in right atrium and passes the interatrial defect arriving directly in cerebral vascularization bypassing lung filter. Considering that the average of PFO dimension is about 9.9 mm, it is sufficient to let pass thrombi of 3 mm or less (1 mm) able to occlude the middle cerebral artery or its branches, respectively [4]. This is supported by evidences showing RLS grade and PFO size as risk factors of stroke and case reports of thrombi localized in PFO tunnels and stroke episodes after the evidence of Deep Venous Thrombosis (DVT) [26, 27]. However, there is no many data supporting the increase of evidence of DVT and stroke in patients with PFO compared to non-PFO patients. Additionally, there are piece of evidence that relate smaller shunts to higher incidence of stroke [28]. Thus, other pathophysiological mechanism and explanation should be implicated in stroke caused by PFO.

To stratify the likelihood of paradoxical embolism the Risk of Paradoxical Embolism (RoPE) score is used [Table 1]. Higher RoPE indicate a larger attributable risk of stroke. Moreover, RoPE score is able to estimate the 2-year risk of recurrence. The calculator considers different variables: history of hypertension, history of diabetes, history of stroke or TIA, smoke habit, presence of cortical damage on imaging and age. To each variable is attributed a score. Basing on the obtained final score, from 0 to 10, likelihood of stroke can be evaluated. Rope from 0 to 3 estimates a risk of 0% but a 20% of recurrence, 9–10 points estimates 88% of risk and a 2% of recurrence [29].

Another potential mechanism of PFO-related stroke is the in situ clot formation [28, 30], probably due to the low flow generated in this area. In particular, specific PFO features, such as length of PFO tunnel, presence of ASA (septum primum excursion ≥10 mm from the plane of the atrial septum into right or left atrium), Chiari’s network or presence of Eustachian valve [31], has been shown to be related to an increase risk of stroke [32].

Intriguingly, arrhythmias have been presumed to be part of the range of hypotheses of PFO-related stroke mechanism. In fact, several investigators seem to support the theory that atrial arrhythmias as atrial fibrillation (AF) contribute to the pathophysiological mechanism of PFO-related stroke, especially when ASA occurs [33].

**Figure 1.**
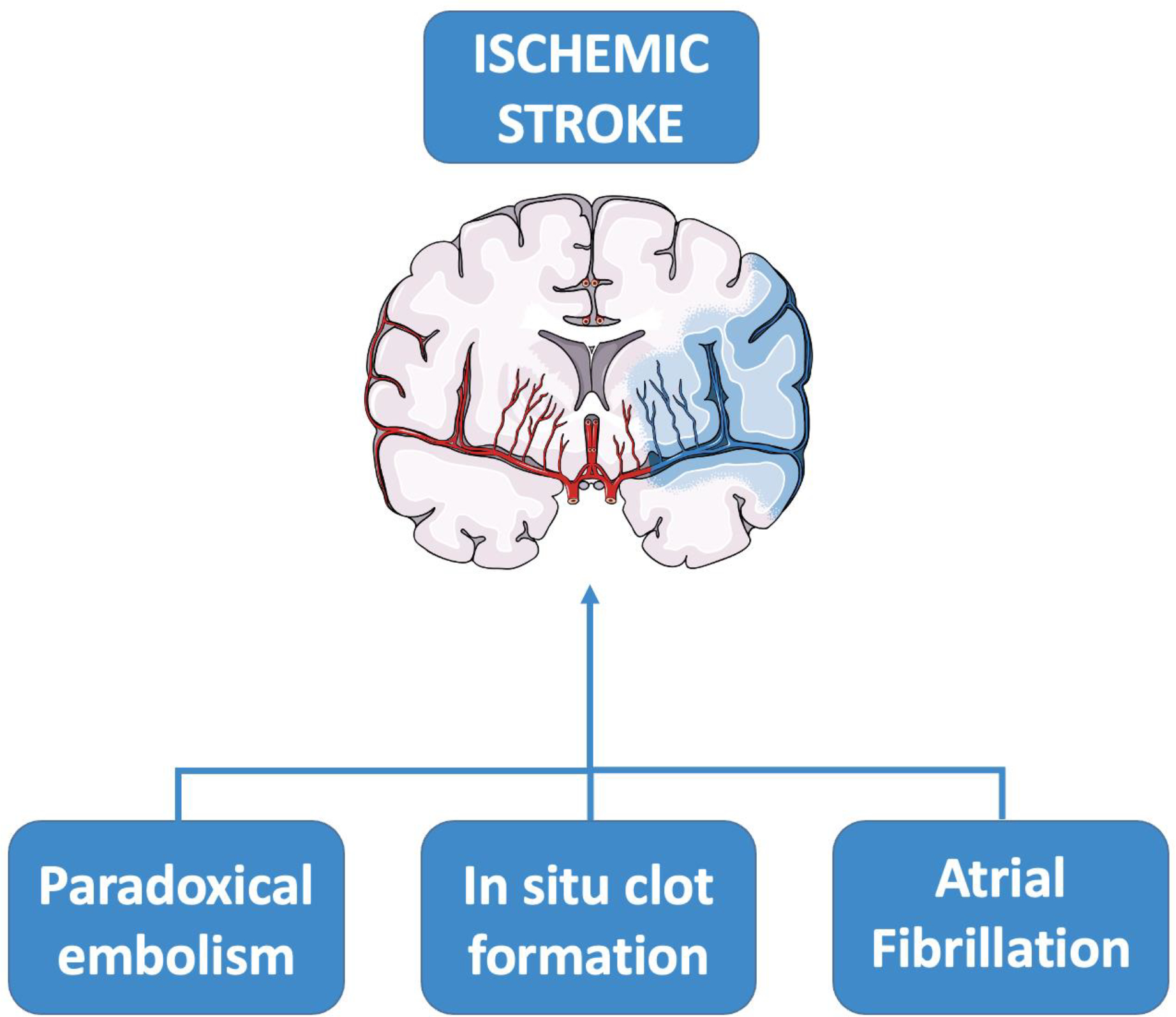
Schematic representation of the pathophysiology of PFO-associated stroke.

Therefore, there are different pathophysiological mechanism that could be involved in PFO-related stroke and each of them can contribute to generate a cerebral ischemic event in young patients with PFO [34] [Figure 1].

### 3.2 Platypnea-orthodeoxia syndrome

Platypnea-orthodeoxia syndrome (POS) is a rare clinical entity characterized by a positional dyspnea and desaturation or hypoxemia when passing from supine to orthostatic position. POS occurs especially in elderly patients, probably because in older age the Eustachian valve is more prominent and the blood coming from inferior vena cava is redirected directly onto the PFO [6]. Desaturation is defined as a drop in PaO2 > 4 mmHg and/or SpO2 > 5% in the passage from supine to orthostatic position. Symptoms relief when the patient lies down [35]. The rationale of these symptoms are the hypoxemia that has supposed to be due to the mixing of the oxygenated blood with the deoxygenated blood through an interatrial defect such as PFO and ASA. Interatrial shunts are not the only cause of this rare clinical entity, shunt could be extra cardiac or the mechanism could be a mix of both. Anyway, the most frequent cause of POS is PFO [22].

Mirwais and co-workers described a very interesting case report of a 87 old year patient with POS and PFO. The symptomatology reverted after PFO occlusion with an Amplatzer PFO occluder device [36].

### 3.3 Migraine

An association between PFO and migraine, in particular migraine headache with aura (MHA), has been reported [37, 38]. Intriguingly, migraine has been associated to a major risk of major adverse cardiovascular and cerebral events (MACCE) but, above all, to an increased risk of stroke both ischemic and hemorrhagic [39]. Evidence shows that in patients with migraine and PFO, transcatheter PFO closure leads to a significant reduction of migraine episodes [40, 41], above all in patients with MHA [42].

Daniela Trabattoni and collaborators discovered a prothrombotic phenotype and even an altered oxidative stress status due to the elevated number of activated platelets in patients with MHA and PFO [43]. The Authors showed a reversion of both conditions after PFO closure in all patients. However, the precise pathophysiological mechanism that link MHA and PFO have not been completely explained [43] but this hypothesis could explain the correlation that some authors found between P2Y12 inhibitors and relief from MHA symptoms [44].

### 3.4 Decompression sickness

Decompression sickness (DCS) is a condition that is observed when there is exposure to hypobaric environment such as returning to sea level after diving. DCS consists in paradoxical embolization of nitrogen bubbles [45]. Clinical manifestation is comprehensive of a wide range of clinical condition from skin rash to severe neurological impairment. Analysis conducted among patients that practice diving and other sports but no diving, showed that cerebral lesions are present more in patient with concomitant PFO. Although, it is necessary to underline that divers present more brain lesions than non-divers independently from presence of PFO [46].

Honek and collaborators have demonstrated that high grade PFO is an independent risk factor for unprovoked DCS in divers; moreover, data from the DIVE-PFO registry have shown that transcatheter PFO closure is more effective in DSC prevention than the conservative approach in divers [47, 48].

## 4. Management

**Figure 2.**
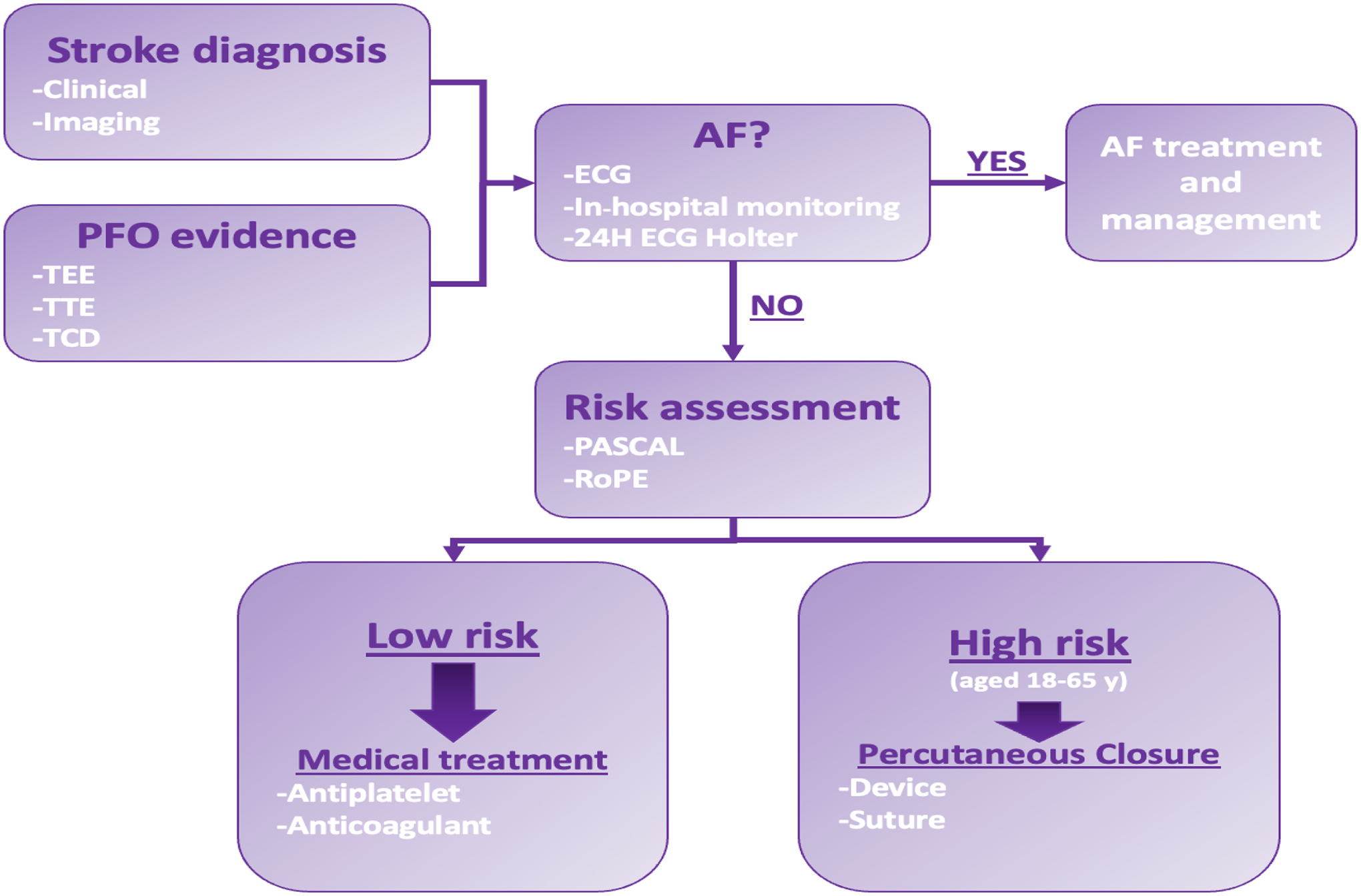
Simplified flow chart of PFO-associated stroke management. AF: Atrial Fibrillation; ECG: Electrocardiogram; PASCAL: PFO-associated stroke causal likelihood system; RoPE: Risk of Paradoxical Embolism score; TCD: TransCranial Doppler; TEE: TransEsophageal Echo; TTE: TransThoracic Echo; y: years.

The PFO management involves a systematic approach to diagnosis, risk assessment, and treatment selection [Figure 2]:

### 4.1. Diagnostic Evaluation:

Correct management of PFO begins with accurately diagnosing interatrial defects using imaging techniques such as transesophageal echocardiography (TEE), transthoracic echocardiography (TTE), transcranial Doppler (TCD), and intracardiac echocardiography (ICE) [12].

### 4.2. Cryptogenic Stroke Assessment:

In cases of cryptogenic stroke with evidence of PFO, it is necessary to analyze the relationship between stroke and PFO to determine causality. This involves excluding differential diagnoses like atrial fibrillation (AF) through a comprehensive diagnostic workup, including electrocardiography (ECG), in-hospital telemetry, and 24-hour Holter-ECG monitoring [2].

### 4.3. Risk Stratification:

Various risk stratification systems, such as the PFO-associated stroke causal likelihood (PASCAL) system [Table 2], aid in stratifying patients based on PFO characteristics and the Risk of Paradoxical Embolism (RoPE) score. These tools help guide treatment decisions, with higher-risk patients potentially benefiting more from PFO closure [11, 29, 49].

### 4.4. Medical Therapy:

Antiplatelet or anticoagulant medications may be initiated for secondary prevention of cryptogenic stroke, particularly in patients with a low or uncertain risk of recurrence. However, the choice between anticoagulation and antiplatelet therapy lacks clear evidence from randomized clinical trials [11, 50, 51].

### 4.5. PFO Closure:

Recurrent stroke attributed to PFO is a clear indication for PFO closure. Recent trials have demonstrated the superiority of transcatheter closure using double-disk design devices over medical therapy alone in preventing stroke recurrence in selected patients [52–58] [Table 3].

### 4.6. Considerations for Elderly Patients:

There is a gap in evidence regarding PFO closure in patients over 60 years old, primarily due to the higher prevalence of comorbidities such as AF. Current guidelines do not recommend routine PFO closure in this age group, emphasizing the importance of thorough cardiac follow-up to detect potential AF [2, 3, 59].

### 4.7. AF Monitoring:

AF is a significant concern following PFO closure, with studies suggesting a notable incidence of post-procedural AF episodes. Careful patient selection is crucial, and monitoring for AF post-procedure is essential for appropriate management [60, 61].

### 4.8. Device Thrombosis Management:

Device thrombosis is a rare but serious complication of PFO closure. Optimal medical therapy post-procedure includes dual antiplatelet therapy (DAPT) for 1–6 months followed by single antiplatelet therapy (SAPT) for 5 years, although further research is needed to refine antiplatelet therapy protocols [62, 63].

### 4.9. Alternative Closure Techniques:

Percutaneous transcatheter suture closure may be considered in selected cases where device closure is not feasible. This technique has shown efficacy and safety in certain anatomical configurations [64–66].

## 5. Conclusions

Managing PFO requires careful consideration of diagnostic findings, stroke risk, and treatment options. While recent trials have supported the efficacy of PFO closure in preventing stroke recurrence, several unresolved questions remain, particularly regarding the optimal management of elderly patients and the prevention of device-related complications. Further research and robust clinical trials are needed to address these gaps and refine the management of PFO for better patient outcomes.

## Figures and Tables

**Table 1.** RoPE score calculator to stratify the likelihood of paradoxical embolism; basing on the obtained final score likelihood of stroke can be evaluated. Maximum score: 10; minimum score: 0. RoPE 0–3 pt: risk 0%, recurrence 20%; RoPE 9–10 pt: risk 88%, recurrence 2%. RoPE: Risk of Paradoxical Embolism; Pt: points; y: years.

CHARACTERISTICS	POINTS
No history of hypertension	1
No history of diabetes	1
No history of stroke/TIA	1
Non smoker	1
Cortical infarct on imaging	1
Age 18–29 y	5
Age 30–39 y	4
Age 40–49 y	3
Age 50–59 y	2
Age 60–69 y	1
Age ≥ 70 y	0

**Table 2.** The PASCAL system divides patients in 3 categories: PROBABLE, POSSIBLE, UNLIKELY. The classification is based on two domains: high-risk PFO (presence of a large shunt with >20 to 30 bubbles, presence of ASA or both, mostly evaluated with TEE) and the RoPE score (23864310). PROBABLE category presents high-risk PFO and RoPE ≥ 7; POSSIBLE category presents high-risk PFO and a RoPE score <7 or low-risk PFO and a RoPE score ≥7; UNLIKELY category presents low-risk PFO and a RoPE score <7. Moreover, PASCAL system estimates risk of development late AF. This risk is based on the hypothesis of occult AF in these patients and on the greater susceptibility to arrhythmogenic effects of device-tissue contact post-implant. Considering a median of 4.8 years of follow-up for each category, there is a rate of increased risk of AF: PROBABLE has a nonsignificant 0.7% increase, POSSIBLE has a 1.5% increase and UNLIKELY has a 4.4% increase in risk of late AF with PFO closure. Thus, PROBABLE and POSSIBLE categories of PASCAL classification are associated with a clear benefit from PFO closure while UNLIKELY PASCAL classification is associated with net harm from closure.

PASCAL CATHEGORY	HIGH-RISK PFO	RoPE score
PROBABLE	Yes	≥ 7
POSSIBLE	(1) Yes / (2) No	(1) < 7 / (2) ≥ 7
UNLIKELY	No	< 7

**Table 3.** Summary table of the clinical trials that evaluated the effectiveness and safety of PFO-percutaneous closure with device.

CLINICAL TRIAL (year)	Number of patients	DEVICE USED	CONTROL ARM	PRIMARY ENDPOINT	P-VALUE (P)	MAIN RESULTS
CLOSURE I (2012)	909	STARflex	Aspirin and/or Warfarin	Composite of stroke/TIA, all-cause mortality, death from neurological causes	HR 0.78 95% CI 0.45 to 1.35 P=0.37	Closure not superior to medical therapy
PC trial (2013)	414	Amplatzer PFO Occluder	Antiplatelet therapy or OAC	Composite of death, nonfatal stroke, TIA, or peripheral embolism	HR 0.63 95% CI 0.24 to 1.72 P=0.34	Closure not superior to medical therapy
RESPECT (2013)	980	Amplatzer PFO Occluder	Aspirin or warfarin or Clopidogrel, or Aspirin with extended release dipyridamole	Composite of recurrent nonfatal ischemic stroke, fatal ischemic stroke or early death after randomization	HR 0.49 95% CI 0.22 to 1.11 P=0.08 HR, 0.27 95% CI 0.10 to 0.75 P=0.007	Intention-to-treat-analysis: no significant benefit for closure; As-treated analysis: closure superior to medical therapy
RESPECT (Long-term follow-up) (2017)	980	Amplatzer PFO Occluder	Aspirin or Warfarin or Clopidogrel, or Aspirin with extended release dipyridamole	Composite of recurrent nonfatal ischemic stroke, fatal ischemic stroke, or early death after randomization	HR 0.55 95% CI 0.31 to 1.0 P=0.046 HR 0.38 95% CI 0.18 to 0.79 P=0.007	Extended follow-up in intention-to-treat analysis: closure superior to medical therapy
CLOSE (2017)	663	CE marked PFO devices	Aspirin or Clopidogrel or Aspirin with extended release dipyridamole/Vitamin K antagonists or NOACs	Recurrent fatal or nonfatal stroke	Closure vs. antiplatelet therapy HR 0.03 95% CI 0 to 0.26 P<0.001/Anticoagulant vs. Antiplatelet therapy HR 0.43 95% CI 0.1 to 1.5 P=0.17	Closure superior to antiplatelet in patients with ASA or PFO with large shunt/Anticoagulant equivalent to antiplatelet therapy
REDUCE (2017)	664	Helex Septal Occluder and Cardioform Septal Occluder	Aspirin or Clopidogrel or Aspirin with dipyridamole	Recurrent stroke/New brain infarct inclusive of silent brain infarct	HR 0.23 95% CI 0.09 to 0.62 P=0.002 HR 0.51 95% CI 0.29 to 0.91 P=0.04	Closure superior to antiplatelet therapy
DEFENCE- PFO (2018)	120	Amplatzer PFO Occluder	Aspirin or Aspirin and Clopidogrel, or Aspirin and Cilostazol, or Warfarin	Stroke, vascular death or TIMI-defined major bleeding	P=0.023 Log-rank P=0.013 P=0.24	Closure in patients with high risk PFO characteristics resulted in lower rate of ischemic stroke vs. medical therapy

ASA: Atrial Septal Aneurysm; CE: *Conformité Européenne*; CI: Confidence Interval; HR: Hazard Ratio; PFO: Patent Foramen Ovale; TIA: transient Ischemic Attack.
